# Undifferentiated Embryonal Sarcoma of the Liver in Pediatrics: A Diagnostic Challenge With Implications for Early Management

**DOI:** 10.7759/cureus.44923

**Published:** 2023-09-08

**Authors:** Amaranto Suárez, Maria Camila Suaza Vallejo, Javier Brito, Juan Pablo Luengas, Jorge Mesa

**Affiliations:** 1 Pediatric Oncology, Instituto Nacional de Cancerología, Bogotá, COL; 2 Pediatric Oncology, Instituto Nacional de Cancerología, Bogota, COL; 3 Hematology and Oncology, Instituto Nacional de Cancerología, Bogota, COL; 4 Pathology, Instituto Nacional de Cancerología, Bogotá, COL

**Keywords:** pediatrics, embryonal liver sarcoma, liver tumor, sarcoma, hepatic sarcoma

## Abstract

A 10-year-old boy was evaluated for intermittent colicky abdominal pain, general malaise, and asthenia. Imaging revealed a solid liver lesion. Subsequent biopsy and extension studies diagnosed the lesion as undifferentiated embryonal sarcoma of the liver, classified as PRETEXT II, group III according to the postoperative staging system of the Intergroup Study for Soft Tissue Sarcomas. He underwent neoadjuvant chemotherapy using alternating cycles of ifosfamide, doxorubicin, vincristine, D-actinomycin, and cyclophosphamide. This was followed by surgical intervention and two additional adjuvant chemotherapy cycles, resulting in a complete disease response. The patient remains in follow-up and shows no signs of relapse 28 months post-diagnosis. Undifferentiated embryonal sarcoma of the liver is a rare and often misdiagnosed condition that can be mistaken for a benign disease. Its prognosis hinges on timely and accurate diagnosis, which is essential for effectively treating patients with this aggressive pathology with a high mortality risk. Notably, there is no standard treatment approach. In our case, we implemented therapeutic strategies from various literature reports, yielding a promising outcome and positive patient progression.

## Introduction

Undifferentiated embryonal sarcoma of the liver (UESL) is a rare tumor of mesenchymal origin first identified in 1978 by Stocker and Ishak [1]. Though it accounts for less than 1% of pediatric cancers, UESL is the third most common primary malignant liver tumor in children behind hepatoblastoma and hepatocellular carcinoma, making up 6% to 13% of liver tumors in this group [2]. Most cases appear in children aged 7 to 13, with no noted sex predominance [2]. Its clinical presentations vary widely from being asymptomatic to symptoms such as abdominal pain, nausea, fever, anorexia, and even severe complications like abdominal bleeding [1]. Due to its aggressive nature, prompt diagnosis and comprehensive treatment are vital for improved survival outcomes [3]. We present a case of a young boy with UESL, initially misdiagnosed due to its atypical presentation, highlighting the diagnostic challenges and subsequent management strategies employed.

## Case presentation

**Figure 1:**
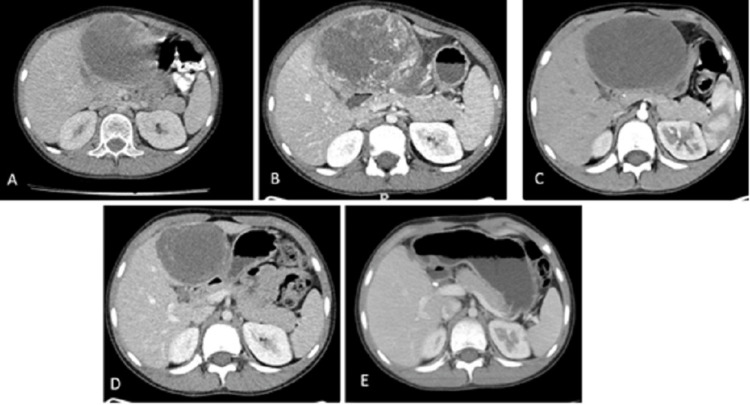
Tomographic evolution in the axial axis of the abdomen. A. Extrainstitutional image at the time of diagnosis: exophytic lesion located in segment IV measuring 87 x 75 mm, partially compressing the gallbladder, contacting its wall without infiltration. B. CT scan performed upon admission to the institution reports a lesion with dimensions of 103 x 85 mm located in segment IV. C. CT scan performed after receiving 2 cycles of ifosfamide/doxorubicin chemotherapy: predominantly liquid mass located in hepatic segment IV, measuring 95 x 69 mm.  D. CT scan performed after 4 cycles of systemic chemotherapy (2 cycles of ifosfamide/doxorubicin and 2 cycles of VAC): Hepatic lesion in segment IV with dimensions of 72 x 60 mm. E. CT scan performed as end-of-treatment evaluation after complete local surgical control and 2 additional cycles of adjuvant chemotherapy: post-surgical changes without evidence of neoplastic involvement.

A 10-year-old boy with no notable medical background came to us, reporting a month of generalized discomfort, weakness, lethargy, and sporadic colicky abdominal pain. An abdominal ultrasound detected a primarily solid liver mass, suggesting either a hepatic hemangioma or sarcoma. A contrast-enhanced abdominal computed tomography (CT) scan displayed an exophytic lesion in hepatic segment IV, sized 87 x 75 mm on the axial plane. This lesion partially pressed against the gallbladder without invading it and showed clear suprahepatic and main portal veins (Figure 1A). A subsequent biopsy identified the lesion as an undifferentiated embryonal hepatic sarcoma, prompting a referral for advanced care.

Upon assessment, the boy was fever-free, and an 8-cm tender mass was palpable in his right upper quadrant. The complete blood count and liver function tests were within reference ranges. Both α-fetoprotein and β-human chorionic gonadotropin levels were within normal limits, and there was no sign of infectious diseases. A follow-up abdominal ultrasound revealed a growth in the right hepatic lobe due to a mainly solid mass showing peripheral flow on Doppler and dispersed hyperechoic areas without acoustic shadowing in hepatic segment IV. This lesion had an exophytic profile with numerous vessels and arteriovenous malformations, suggesting a sizable hemangioma. Another contrast-enhanced abdominal CT depicted a lesion in hepatic segment IV, measuring 103 x 85 mm, with increased fatty density nearby in the peritoneum and slight compression of the gallbladder without invasion (Figure 1B).

Our institution's pathology department review described a malignant tumor consisting of spindle and pleomorphic cells with vague cytoplasm. These cells were organized in relaxed sheets with some hemorrhagic necrosis zones. Immunohistochemical tests indicated positive results for cluster of differentiation 56 (CD56), vimentin, B-Cell lymphoma 2, and desmin, with a proliferation rate (as measured by the KI67 index) of 60%. The tumor did not react to myogenic differentiation, myogenic factor 4, glypican 3, or keratin AE1-AAE3, confirming the diagnosis of undifferentiated embryonal hepatic sarcoma.

Considering the extensive involvement of liver segments I, III, and IV and the tumor size that initially rendered a full resection infeasible, the patient underwent neoadjuvant chemotherapy with ifosfamide (3 g/m2/day on Days 1, 2, and 3 of each cycle) and doxorubicin (37.5 mg/m2/day on Days 1 and 2 of each cycle), administered every 21 days. After two cycles, a subsequent CT scan showed a mostly liquid mass in hepatic segment IV, measuring 95 x 69 mm on the axial plane, with the stable disease indicated (Figure 1C). After receiving additional chemotherapy with vincristine (1.5 mg/m2/day on Day 1 of each cycle, maximum 2 mg), D-actinomycin (0.045 mg/kg/day on Day 1 of each cycle, maximum 2.5 mg), and cyclophosphamide (1.2 g/m2/day on Day 1 of each cycle) every 28 days, completing two cycles, a subsequent abdominal CT scan displayed a 75% size reduction of the hepatic lesion in segment IV compared to the first scan, with no signs of infiltration into nearby organs (Figure 1D).

**Figure 2:**
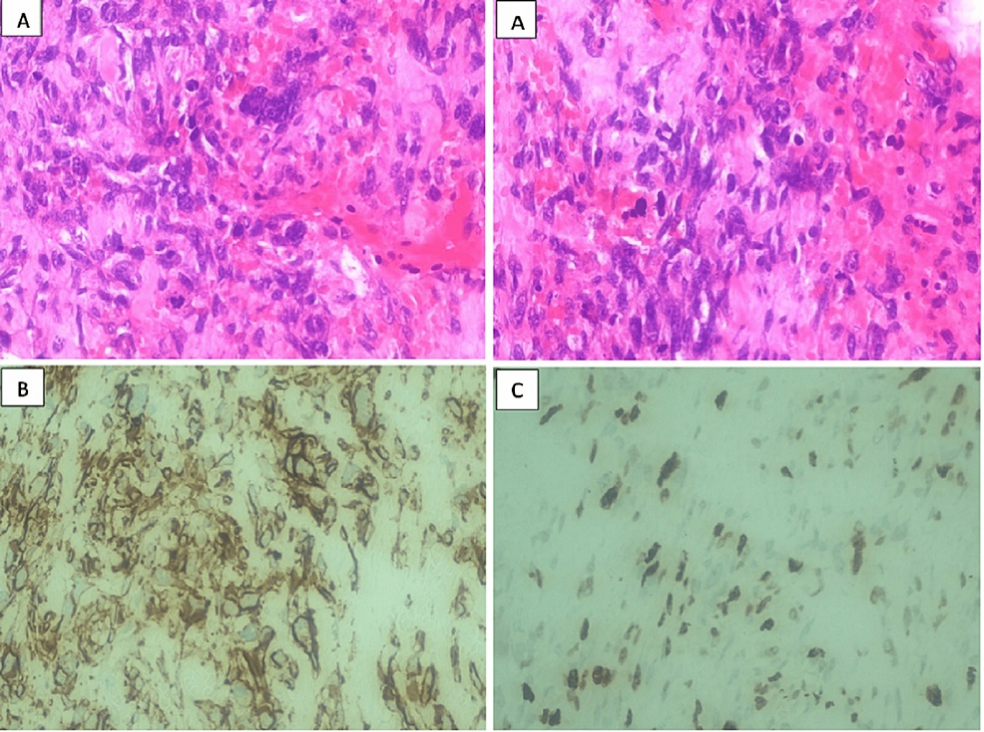
Histopathology A. 100X H&E Stain, Malignant tumor composed of spindle-shaped and pleomorphic cells, with poorly defined cytoplasm, arranged in loose sheets, post-treatment condition, 99% tumor necrosis. B. Immunohistochemistry: Strong membrane staining observed in tumor cells with CD56. C. KI67 index of 40%.

A surgical oncologist then assessed the boy and performed a resection using intraoperative ultrasound guidance. This procedure involved an extra-anatomic segmentectomy of segments IV and part of V, including the gallbladder in the resection. Post-treatment pathology confirmed the presence of undifferentiated embryonal hepatic sarcoma, measuring 75 x 72 mm, with 1% viable tumor and 99% necrosis. The tumor displayed positive markers for glypican 3, vimentin, CD56, and desmin, with a KI67 index of 40%. No tumor was detected at the section margins (Figure 2).

The patient began adjuvant chemotherapy 15 days post-resection and completed six cycles. Post-treatment imaging, including an abdominal CT, showed changes resulting from the hepatic segment IV resection but no masses at the surgical margin. The remaining liver segments appeared normal, with no detected lesions (Figure 1E). A contrast-enhanced chest CT also displayed no deviations from prior images or concerning mediastinal lesions. As of this writing, 28 months post-initial diagnosis, the patient remains healthy during the follow-up phase and has shown no signs of disease progression. 

## Discussion

UESL is an infrequent and aggressive malignant tumor originating from primary mesenchymal tissue [4]. It represents 1% of all new cancer diagnoses in the United States. Among hepatic tumors in children, UESL ranks third, predominantly affecting children under 15 years old (88% of cases). Stocker and Ishak first characterized it as a hepatic mesenchymal tumor in 1978 [1,5]. The etiology and pathogenesis remain incompletely elucidated. Nevertheless, an association exists between UESL and mesenchymal hamartoma, given their shared cytogenetic anomalies. Both conditions are often considered part of the same disease spectrum [3,4]. Recent studies suggest that mesenchymal hamartoma might transform malignantly, as indicated by overlapping clinical, pathological, and cytogenetic features, particularly aberrations on chromosome 19q13 [3-5]. Other noted chromosomal abnormalities include amplifications or deletions on chromosomes 1q, 5p, 8p, and 12q [6], with aberrations in chromosomes 6, 11, 12, X, and mutations in p53 also reported [7].

Clinically, UESL may be detected unexpectedly in an asymptomatic child or might present symptoms such as an abdominal mass, pain, fever, weight loss, and anorexia, like in our case. Other symptoms can include vomiting, diarrhea, lethargy, constipation, and dyspnea [3,4]. Fever is often linked to tumor-associated hemorrhage and necrosis. The spontaneous rupture of the tumor due to rapid growth is infrequent but can lead to hemorrhage in the abdominal cavity [4,8,9]. Some cases report extrahepatic dissemination [4,5], with metastases identified in 5% to 13% of cases, commonly targeting the lungs and lymph nodes, followed by the pleura and peritoneum [10].

There are no unique serum markers for UESL. Some patients may show signs of leukocytosis and anemia potentially resulting from intratumoral events like hemorrhage, necrosis, or infection [4]. Occasionally, elevated levels of α-fetoprotein and cancer antigen 125 have been recorded [4,5]. Only elevated lactate dehydrogenase was noted in our patient, with 71% of patients with UESL showing similar findings [4].

On imaging, X-rays may depict dense, soft tissue in the upper right quadrant with the displacement of adjacent intestinal loops. Ultrasound often reveals a primarily solid, encapsulated tumor with varied echogenicity. Typically, tumors exceed 10 cm at diagnosis [11]. CT may showcase mixed cystic-solid lesions with delayed enhancement [4]. However, a known paradoxical appearance arises when ultrasound shows a solid tumor, while CT or magnetic resonance imaging (MRI) displays a cystic character. This discrepancy arises due to the high water content in the myxoid stroma [5]. MRI can show various features depending on tumor characteristics and sequences [1,9,11]. These radiological findings can mislead practitioners, mimicking benign hepatic lesions [3]. Positron emission tomography or CT with 18-fluorodeoxyglucose has emerged as a valuable tool for staging, monitoring treatment response, and watching for recurrence. Nonetheless, it is imperative to minimize radiation exposure, especially in pediatric patients [11].

UESL diagnosis is confirmed via histopathology and immunohistochemistry [4]. Typically, macroscopic examination unveils a solitary, clear-cut lesion encased by a fibrous pseudo-capsule formed by the surrounding liver tissue. The tumor predominantly appears in the right hepatic lobe [9] and often ranges between 10 and 30 cm upon diagnosis. Microscopic features and immunophenotype patterns can vary [3,4].

Due to UESL's rarity and its potential for misdiagnosis, many patients receive inaccurate preoperative evaluations. Several differential diagnoses exist, ranging from benign conditions like hepatic mesenchymal hamartoma and hydatid cysts to more severe diseases like hepatoblastoma and hepatocellular carcinoma [1,3,9].

Treatment strategies have evolved over the years. Initially, treatment centered on surgical resection, yielding survival rates below 40% [2-4]. Subsequent incorporation of multimodal approaches based on established guidelines improved survival outcomes. The Cooperative Weichteilsarkom Studie (CWS) and the Italian Soft-Tissue Sarcoma Study (ICG) adopted a multimodal strategy for UESL treatment based on the IRSM (Inter-group Rhabdomyosarcoma Study) guidelines, resulting in improved overall survival rates at five years, ranging from 70% to 100% [2, 11-13]. While surgery remains pivotal, achieving complete resection with negative margins can be challenging. Combining neoadjuvant chemotherapy, surgery, adjuvant chemotherapy, and occasionally radiation offers the best chance for cure [12-14]. Different chemotherapy regimens have been reported, including alkylating-based regimens, VAC (vincristine, dactinomycin, and cyclophosphamide), IVA (ifosfamide, vincristine, and actinomycin-D), and CAVAIE (carboplatin, epirubicin, vincristine, actinomycin-D, ifosfamide, and etoposide) [15-18], with five-year survival rates ranging from 70% to 90% in these cases. Despite various chemotherapy regimens, a definitive consensus remains elusive. Our patient underwent chemotherapy followed by successful surgical resection, eliminating the need for radiation. The exact role of radiation in UESL management, especially in recurrence prevention and survival enhancement, remains debatable [2,13-15]. For those with specific complications or disease progression, liver transplantation has been considered as an alternative [1,4,13]. The prognosis varies, and while several determinants have been suggested, further research is required to establish definitive predictors [2,10,12].

## Conclusions

UESL, though rare, poses significant clinical challenges due to its aggressive nature and potential for misdiagnosis. This report underscores the necessity for healthcare practitioners, including pediatric specialists, to recognize UESL when evaluating atypical liver lesions in children aged eight to 18. Heightened awareness can expedite accurate diagnosis and timely intervention, improving patient outcomes.
